# Microbial community dynamics during aerobic granulation in a sequencing batch reactor (SBR)

**DOI:** 10.7717/peerj.7152

**Published:** 2019-08-29

**Authors:** Fabiola Gómez-Basurto, Miguel Vital-Jácome, Elizabeth Selene Gómez-Acata, Frederic Thalasso, Marco Luna-Guido, Luc Dendooven

**Affiliations:** 1 Laboratory of Soil Ecology, Cinvestav, Mexico City, Mexico; 2 Laboratory of Bioprocesses, Cinvestav, Mexico City, Mexico

**Keywords:** Sequencing batch reactor, Microbial community diversity, Aerobic granules

## Abstract

Microorganisms in aerobic granules formed in sequencing batch reactors (SBR) remove contaminants, such as xenobiotics or dyes, from wastewater. The granules, however, are not stable over time, decreasing the removal of the pollutant. A better understanding of the granule formation and the dynamics of the microorganisms involved will help to optimize the removal of contaminants from wastewater in a SBR. Sequencing the 16S rRNA gene and internal transcribed spacer PCR amplicons revealed that during the acclimation phase the relative abundance of *Acinetobacter* reached 70.8%. At the start of the granulation phase the relative abundance of *Agrobacterium* reached 35.9% and that of *Dipodascus* 89.7% during the mature granule phase. Fluffy granules were detected on day 43. The granules with filamentous overgrowth were not stable and they lysed on day 46 resulting in biomass wash-out. It was found that the reactor operation strategy resulted in stable aerobic granules for 46 days. As the reactor operations remained the same from the mature granule phase to the end of the experiment, the disintegration of the granules after day 46 was due to changes in the microbial community structure and not by the reactor operation.

## Introduction

The formation of aerobic granules in sequencing batch reactors (SBR) has been used as a technique to remove specific contaminants, such as xenobiotics or dyes, from wastewater (Coma et al., 2012). The functioning of the SBR and granule formation is controlled by the operation parameters (Weissbrodt, Shani & Holliger, 2014). The cycle time and the organic loading rate (OLR) determine the size and characteristics of the granule. A cycle of 1.5 h generated the largest granules whereas 4 h resulted in the most condense ones (Liu & Tay, 2007). An OLR with a value of chemical oxygen demand (COD) of 2.52 kg/m^3^ per day was adequate for granule formation (Kim, Kim & Jang, 2008). Aerobic granules formation from wastewater at an OLR of 1.05–1.68 kg COD/m^3^/day was possible, although 9 months were needed to form mature granules (Wang et al., 2009).

Granulation and the microbial community structure in the SBR are controlled by different factors, such as type of C substrate operation temperature, pH, oxygen level, aeration rate and settling time (De Kreuk, Pronk & Van Loosdrecht, 2005). Different C substrates, for example, glucose, acetate, phenol or ethanol, have been used to produce aerobic granules with distinct morphology and different microbial community structures. The granules produced with glucose as C substrate had a loose structure and filamentous bacteria dominated, while using acetate resulted in more compact granules with less filamentous bacteria (Tay, Liu & Liu, 2001).

The pH of the medium affects the growth of the microorganisms. Fungi might be involved in initial granulation at a low pH and bacteria at higher pH. For instance, Yang, Li & Yu (2008) reported that at pH 3, granules measured 7.0 mm and were dominated by fungi after 1 week, while at pH 8 granules were 4.8 mm and dominated by bacteria after 4 weeks.

Oxygen is the final electron acceptor in aerobic biological processes, and dissolved oxygen (DO) is a decisive factor in activated sludge processes. Low DO values generated filamentous granules with poor settleability that diminished the extracellular polymeric substances production and the nitrifying activity (Hu et al., 2005). Therefore, DO values should be maintained above two mg/L in the reactor.

Aeration rate (shear force) also affected granulation. At low aeration intensity (one L/min), granules are not formed. A high aeration rate (three L/min) rendered compact granules (1–1.5 mm), while intermediate aeration (two L/min), did not result in a sufficient oxygen supply to break down filamentous structures leading to the formation of large granules (3–3.5 mm) and SBR failure (Adav, Lee & Lai, 2007). A short settling time led to a washout of poorly settled suspended solids, while suitable settled granules were retained (Qin, Liu & Tay, 2004).

Bacteria are known to produce extracellular polymeric substances under stress and form a matrix rich in polymers (Sutherland, 2001). The extracellular polymeric substances supported the formation and preserved the morphology of aerobic granules through the attachment of microbial cells (Adav, Lee & Tay, 2008). Consequently, metabolic obstruction of polysaccharide synthesis stopped microbial agglomeration (Cammarota & Sant’Anna, 1998).

In previous studies, a microrespirometric system was used to study the biodegradation 4-chlorophenol by aerobic granules in a SBR. It was found that 4-chlorophenol was removed at 0.9 kg/m^3^ of COD per day (Vital-Jácome et al., 2016) and members of *Sphingobium*, *Comamonadaceae* and *Rhizobiaceae* were enriched during its degradation in the aerobic granules (Gómez-Acata et al., 2018). Thus, a better understanding of the community distribution, structure and stability of the aggregates will help to further understand and optimize granule formation in SBR and will help to improve their use in wastewater treatment. The aim of this work was to investigate the dynamics of the microbial community during the formation, maturation and destabilization of aerobic granules using metagenetics of the 16S rRNA and high throughput sequencing.

## Materials and Methods

### Reactor setup and operation

The SBR used and its operation was mostly the same as reported by Gómez-Acata et al. (2018). Briefly, the reactor was a glass bubble column with 0.12 m internal diameter, a total volume capacity of nine L and a five L working volume. The SBR operation was automated with peristaltic pumps to control wastewater inflow and effluent outflow by precision timers (Masterflex L/S economy drive Cole Palmer, Model 7554). The single bioreactor was operated for 50 days. Three settling times were applied during reactor operation, 30 min during phase I (acclimation), 3 min during phase II (start of granulation), and 1 min during phase III (mature granules) and onwards. The reactor was operated with a reaction phase of 4 h during the first 22 days, equivalent to a residence time of 0.33 days, and a reaction phase of 6 h after day 22, equivalent to a residence time of 0.5 days. Aeration in the reactor was maintained at two L/min during the first 22 days and was increased to 2.5 L/min afterwards. The temperature was controlled at 25 ± 1 °C. The OLR expressed as COD was kept constant during the operation of the reactor at 6.4 ± 0.2 kg/m^3^ day.

### Seed sludge and wastewater composition

The SBR was inoculated with activated sludge from an aerobic wastewater treatment plant located in Mexico City (Mexico, N.L. 19°52′ W.L. 99°15′) with an initial biomass concentration of 4.1 kg/m^3^ COD. The reactor was fed with synthetic wastewater with the following composition: 25.5 mg/L KH_2_PO_4_, 32.6 mg/L K_2_HPO_4_, 75.4 mg/L Na_2_HPO_4_ · 7H_2_O, 8.6 mg/L MgSO_4_ · 7H_2_O, 36.4 mg/L CaCl_2_ · 2H_2_O, 0.25 mg/L FeCl_3_ · 6H_2_O, 0.035 mg/L MnCl_2_ · 4H_2_O, 0.057 mg/L H_3_BO_3_, 0.020 mg/L ZnCl_2_, 0.0347 mg/L (NH_4_)_6_Mo_7_O_24_ and 0.0555 mg/L EDTA. The sole carbon source was sodium acetate supplied to the SBR at a concentration of two kg/m^3^, expressed as COD, during the first 22 days and three kg/m^3^ afterwards, equivalent to an absolute concentration of sodium acetate 2.9 and 4.4 g/L, respectively. The carbon to nitrogen ratio (C/N) was maintained at 10 by adding NH_4_Cl as nitrogen source. The pH was adjusted and controlled at 7 ± 0.15 during the whole experiment.

### SBR parameters

Analytical techniques were used as stipulated in the “Standard Methods for the Examination of Water and Wastewater” (Method 5220; APHA, 1999). Total and soluble COD was measured by the closed reflux method. The total COD was measured from unfiltered samples of the reactor, while the soluble COD was measured from filtered samples (0.2 μm nylon membrane, 66601; Mexico City, Mexico). The concentration of substrate corresponded to the soluble COD and the concentration of biomass corresponded to the difference between total and soluble COD. The concentration of biomass was also determined as the mixed liquor volatile suspended solids (MLVSS) using standard gravimetric methods. The concentration of nitrogen was determined through total nitrogen measurements (Shimadzu TOC-Vcsn equipped with a TNM-1 module; Shimadzu, Fraccion Industrial Alce Blanco, Mexico).

The sludge volume index (SVI) was determined using an Imhoff sedimentation cone. The granule size was defined as the Feret diameter. It is the longest distance between any two points along a selection boundary and also known as maximum caliper (Walton, 1948). It was determined by taking pictures from samples using a digital camera with a supermacro option (Stylus TG-2; Olympus, Miami, FL, USA) and by image analysis using the ImageJ software. During the operation of the reactor, the food to microorganism ratio (F/M), the COD and the nitrogen removal efficiency were monitored. Additionally, the DO concentration was measured continuously using a polarographic oxygen probe (HI2400; Hanna Instruments, Woonsocket, RI, USA) located at a medium height to determine the reactor performance during each SBR cycle.

### DNA extraction, PCR and sequencing

Three 50 mL samples were taken from the middle of the SBR on days 0, 4, 6, 8, 12, 13, 18, 20, 22, 25, 27, 29, 32, 34, 36, 39, 43, 46 and 50. Samples were centrifuged at 2,900×*g*, 10 °C for 30 min to concentrate the suspended biomass. The DNA was extracted from each sample (*n* = 3) on each day (*n* = 19) as reported previously by Navarro-Noya et al. (2013). Amplicon libraries of V3–V4 regions of 16S rRNA genes were obtained using primers 341F (5′-CCTACGGGIGGCWGCAG-3′) and 805R (5′-GACTACHVGGGTATCTAATCC-3′) (Klindworth et al., 2013) containing the Illumina-specific MID barcodes. The PCR mixture contained 10 pM of each primer, 200 μM of each dNTP, 0.7 U DNA polymerase (Thermo Fisher Scientific, Hudson, NH, USA), 1 × reaction buffer, 0.002 mol/L MgCl_2_ and 10 ng metagenomic DNA in a reaction volume of 20 μL. Amplification was done with the PCR Thermal cycler Multigene Optimax (Labnet, Edison, NJ, USA) using the following steps: (1) initial denaturation at 94 °C for 10 min, (2) 25 cycles of further denaturation at 94 °C for 45 s, annealing at 53 °C for 45 s, and extension at 72 °C for 1 min, (3) concluding extension at 72 °C for 10 min. Fungal internal transcribed spacer (ITS) was amplified using ITS5F (5′-GGAAGTAAAAGTCGTAACAAGG-3′) and ITS4R (5′-TCCTCCGCTTATTGATATGC-3′) (White et al., 1990) containing the Illumina-specific MID barcodes. The PCR amplification was done as mentioned above with a PCR mixture that contained 1.25 μl DMSO. The region V1–V3 of the 16S rRNA gene from Archaea was amplified using 25F (5′-CYGGTTGATCCTGCCRG-3′) (Dojka et al., 1998) and 571R (3′-CTACGGNYSCCTTTARGC-3′) primers (Baker, Smith & Cowan, 2003) containing the Illumina-specific MID barcodes.

The DNA from each sample was amplified three times, pooled, purified using the Fast Gene™ Gel/PCR Extraction Kit (Nippon Genetics Europe GmbH, Tokio, Japan), assessed for integrity by agarose (1%) gel electrophoresis and quantified with a Nanodrop™ 3300 Fluorospectrometer (Thermo Fisher Scientific Inc, Waltham, MA, USA) using Quant-iT^™^ PicoGreenR dsDNA (Invitrogen, Carlsbad, CA, USA). Equal amounts of the purified 16S rRNA gene and ITS (ITS5F–ITS4R region) amplicons were sequenced at Macrogen (Macrogen Inc., Seoul, Korea), using paired-end (2 × 300 nt) Illumina MiSeq sequencing system (Illumina, San Diego, CA, USA).

### Analysis of sequencing data

QIIME version 1.9.1 was used to filter sequences for quality (Caporaso et al., 2010). Scores less than 75% and mismatches in the barcode or in the primers were eliminated from the files. The number of operational taxonomic units (OTUs) was determined with UCLUST algorithm at 97% similarity (Edgar, 2010). Taxonomic assignation of 16S rRNA was done with the Greengenes core-set-aligned with UCLUST (http://greengenes.secondgenome.com/?prefix=downloads/greengenes_database/gg_13_5/) and the UNITE sequence sets for ITS with BLAST (UNITE, 2017). A total of 2,873 OTUs of the 16S rRNA gene and 919 OTUs of the ITS were used in subsequent analysis.

The relative abundance of the phenotypic categories of the taxonomic groups was predicted using METAGENassist, that is, a statistical tool for comparative metagenomic (Arndt et al., 2012). Data filtering was based on interquantile range, row normalization by sum and column normalization based on autoscaling. The metabolic capacity of bacterial communities was predicted using PICRUSt 1.1.1 software (Langille et al., 2013) and the Kyoto Encyclopedia of Genes and Genomes (KEGG). Details can be found in Gómez-Acata et al. (2018). The nearest sequenced taxon index scores of each sample were calculated (Table S1).

Abundance of the bacterial genera was explored with a principal component analysis (PCA) with the vegan package in the R environment (Oksanen et al., 2017). Heatmaps were constructed with the pheatmap package (Kolde, 2015).

### Availability of data and materials

The datasets supporting the conclusions of this article are available in the National Center for Biotechnology Information sequence read archive under the BioProject accession numbers PRJNA419221 and PRJNA422681.

## Results

### Seed sludge

**Figure 1:**
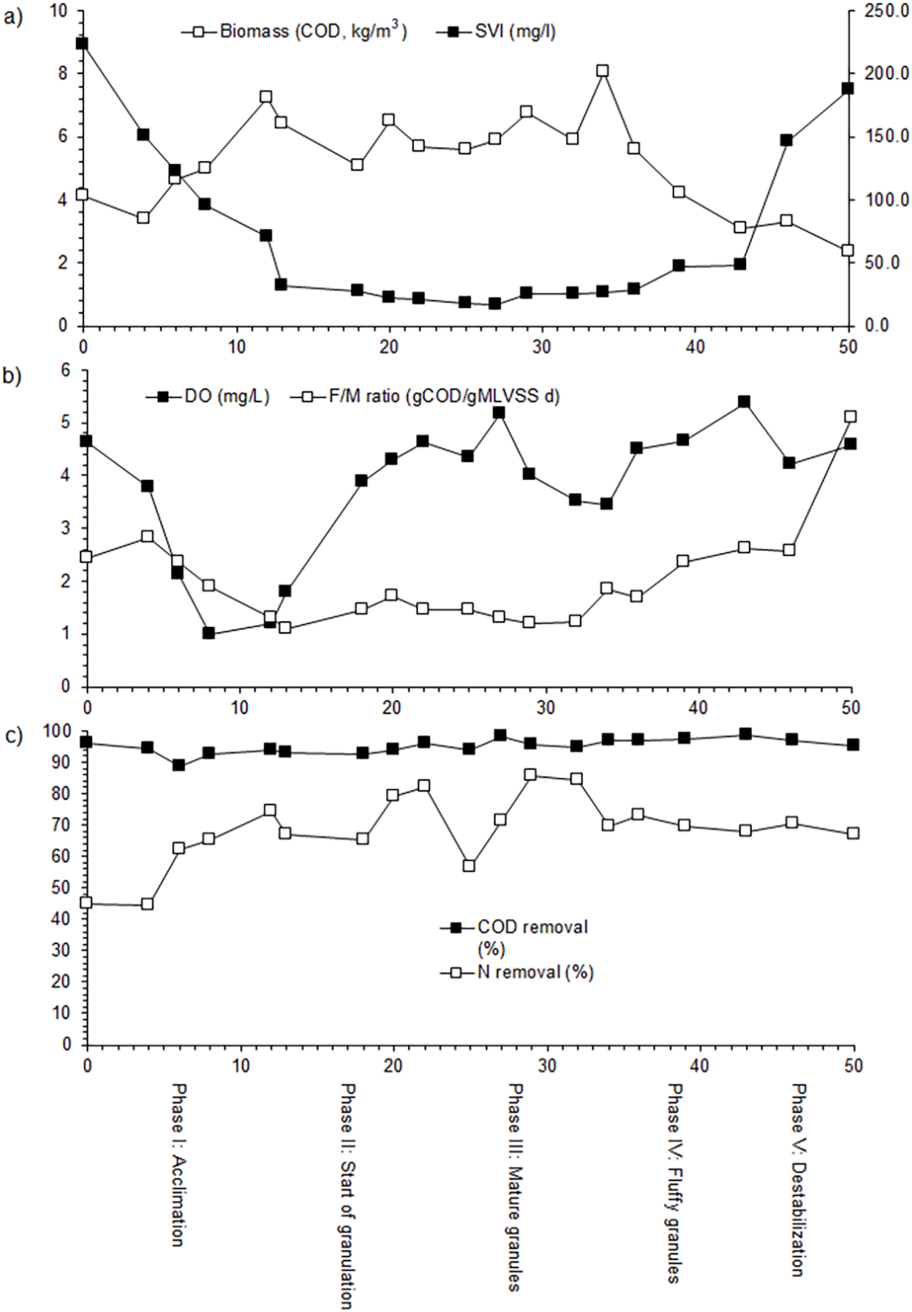
Evolution of the different biological and hydro-chemical parameters, (A) biomass and sludge volume index (SVI); (B) dissolved oxygen (DO) and food to microorganisms (F/M) ratio; (C) chemical oxygen demand (COD) and nitrogen removal, during the SBR operation.

**Figure 2:**
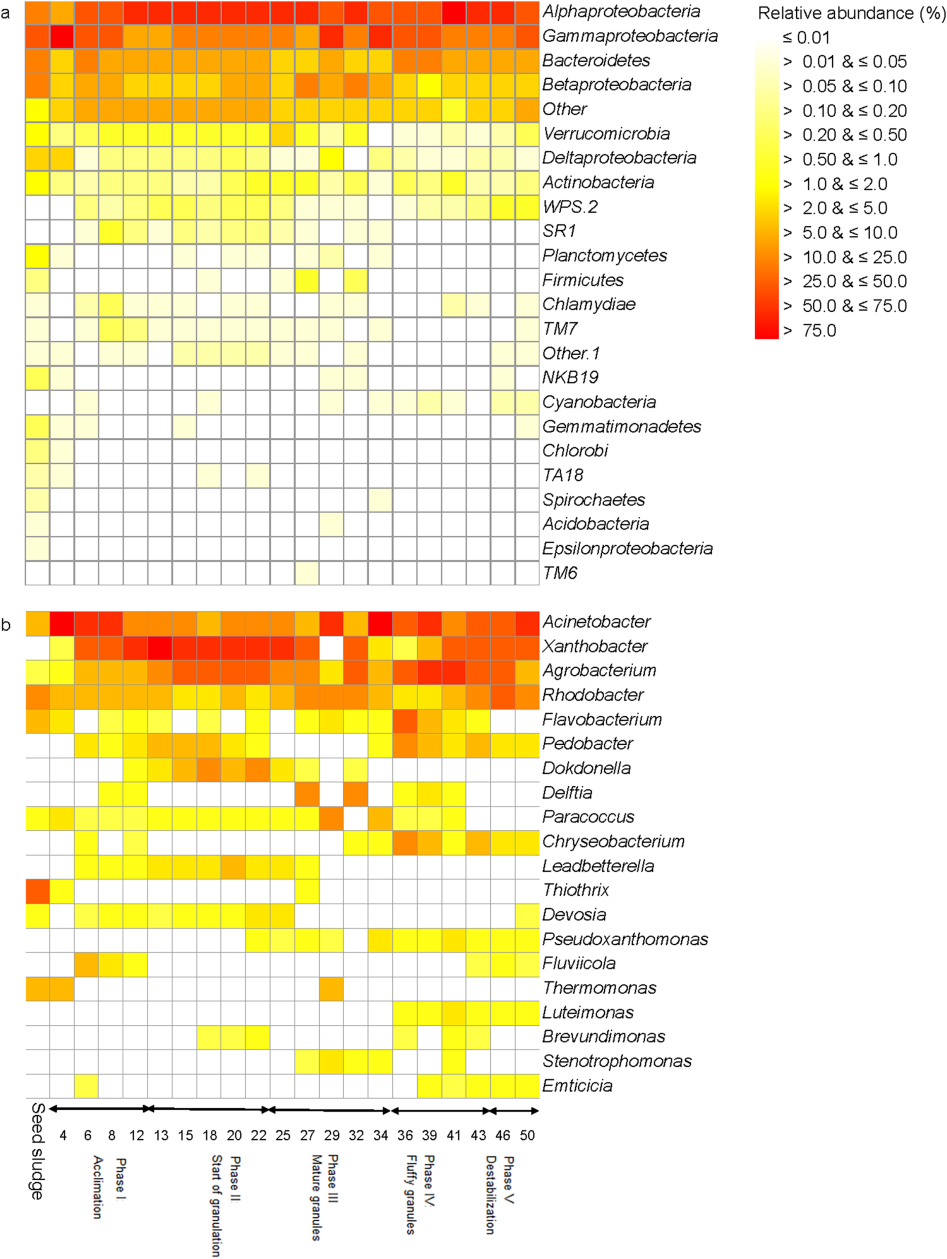
Heatmap with (A) the relative abundance of the bacterial phyla and (B) the 20 most abundant genera as detected in the different phases in the sequencing batch reactor used in this study.

**Figure 3:**
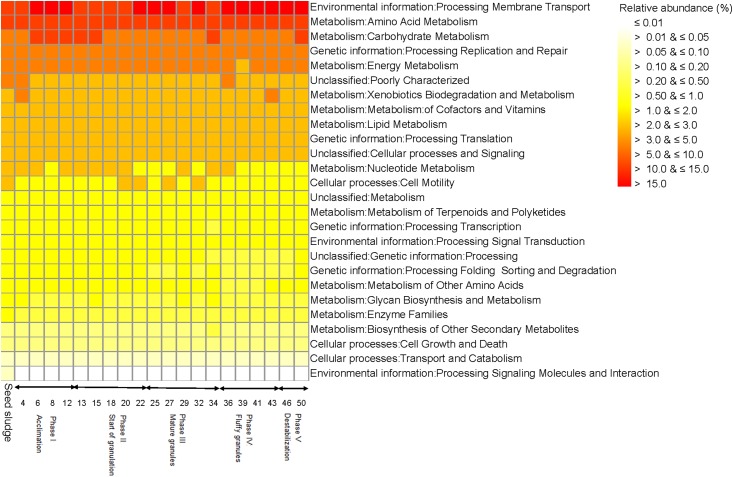
Heatmap with the functionality of bacterial communities predicted with the Kyoto Encyclopedia of Genes and Genomes (KEGG) as detected in the different phases in the sequencing batch reactor used in this study.

The seed sludge collected had a SVI of 222.6 mL/g and a biomass of 4.1 g COD/L (Fig. 1A). Overall, 18 different bacterial phyla were detected in the seed sludge. The *Proteobacteria* were the dominant bacterial phylum with a relative abundance of 72.4% (mostly *Gammaproteobacteria* (42.7%) and *Alphaproteobacteria* (15.7%)) followed by *Bacteroidetes* (21.4%) (Fig. 2A). The most abundant genus was *Thiothrix* (mean relative abundance 10.7%), followed by *Rhodobacter* (5.7%) and *Flavobacterium* (4.7%) (Fig. 2B). The comparative metagenomic analysis predicted that 56.0% of the bacteria in the inoculum had ammonia oxidizing capacity in the seed sludge, 17.4% nitrite reducing capacity, 26.5% sulfate reducing capacity and 24.1% sulfide oxidizing capacity (Table 1). In the seed sludge the most abundant predicted KEGG orthologs were signaling molecules, transport and catabolism (Fig. 3).

**Table 1:** Metabolic activities predicted by METAGENassist program during the sequencing batch reactor (SBR) operation. Values expressed as percentage %.

Phases	Day	Ammonia oxidizer	Denitrifying	Nitrite reducer	Nitrogen fixation	Sulphate reducer	Sulphide oxidizer	Sulphur oxidizer
Seed sludge	0	56.0	0.6	17.4	14.5	26.5	24.1	5.2
I Acclimation	4	85.5	0.7	78.2	6.1	80.5	8.1	73.7
	6	72.7	2.0	58.5	15.4	65.5	15.0	49.9
	8	72.9	3.6	67.3	12.8	72.2	8.2	60.0
	12	78.1	24.5	57.8	38.4	67.7	21.2	25.5
II Start of granulation	13	80.8	27.9	68.5	44.8	77.7	15.1	31.0
	18	75.2	18.3	63.5	54.7	75.0	31.7	34.0
	20	70.5	12.3	50.4	48.9	69.2	34.9	27.3
	22	70.9	4.3	57.1	32.1	70.3	28.3	46.2
III Mature granules	25	69.8	3.0	60.7	27.5	69.2	24.9	48.3
	27	74.7	3.2	66.6	36.6	73.4	33.9	55.6
	29	78.1	4.7	69.2	63.3	75.6	60.5	57.5
	32	81.2	6.8	74.2	70.5	80.6	64.5	62.5
	34	72.7	12.6	64.4	61.6	72.3	50.0	45.7
IV Fluffy granules	36	70.4	14.9	61.5	58.0	70.9	44.4	40.0
	39	69.8	17.2	60.6	53.6	67.0	37.2	34.1
	43	75.4	10.9	69.4	61.0	77.0	49.5	52.1
V Destabilization	46	78.0	9.2	68.2	37.5	75.2	28.4	24.8
	50	82.6	14.6	68.9	47.5	76.2	29.4	24.4

**Figure 4:**
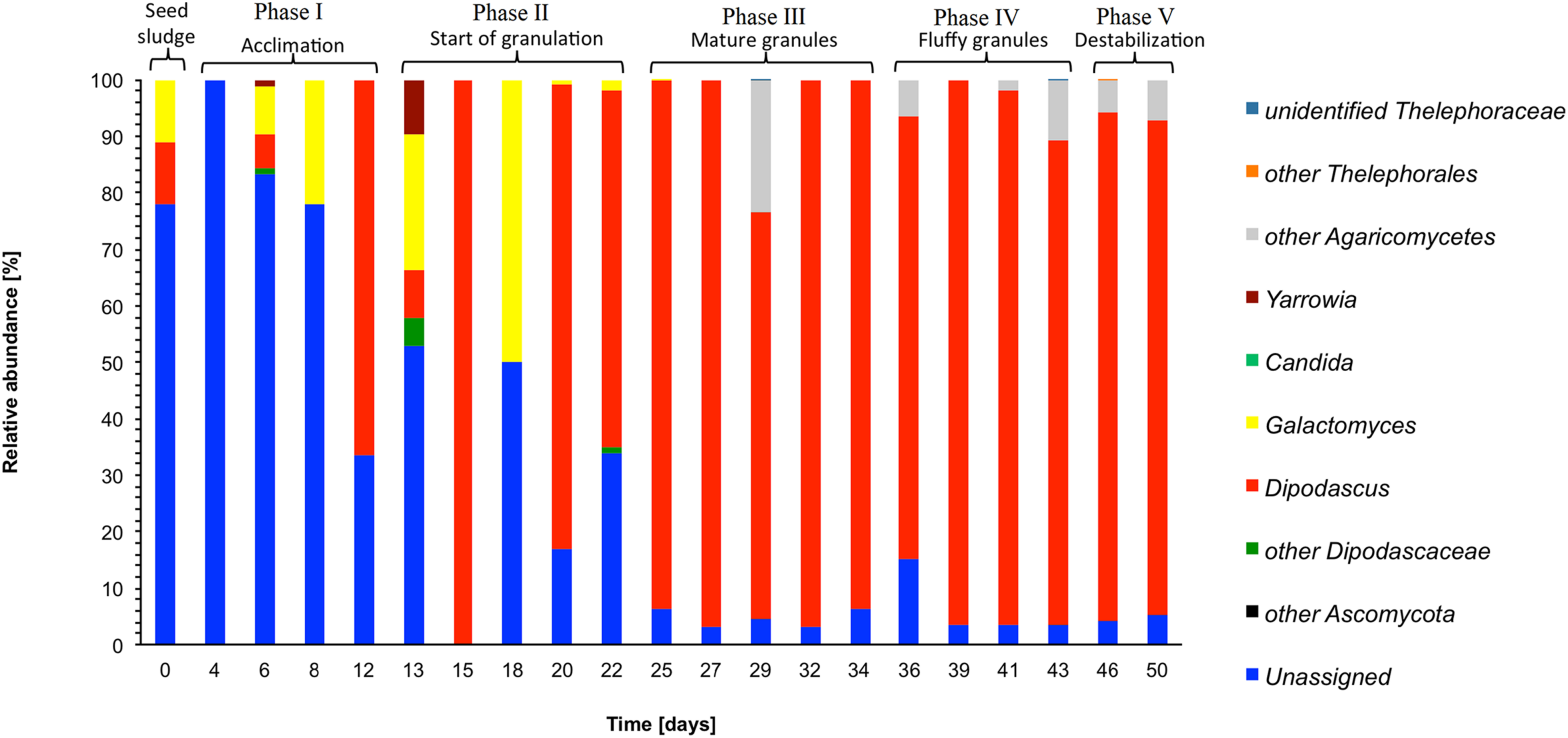
Heatmap with the relative abundance of the fungal genera as detected in the different phases in the sequencing batch reactor used in this study.

In the seed sludge, phylotypes could be assigned to only two fungal genera, that is, *Dipodascus* and *Galactomyces* (Fig. 4). Most phylotypes detected in the seed sludge could not be assigned to a fungal group.

### The SBR operation at settling time 30 min: day 4 to day 12: Phase I, acclimation

Five phases could be defined during the SBR operation. Phase I (acclimation) was defined as the period from the beginning of the experiment to the major change in the operating settling time, that is, when reduced from 30 to 3 min. This sudden change induced the growth of the granules. The other stages were defined by the morphological changes of the granules in the reactor, that is, phase II, the start of the formation of the granules, phase III, the formation of more stable granules, phase IV, the formation of filamentous granules and phase V, destabilization of the granules.

**Figure 5:**
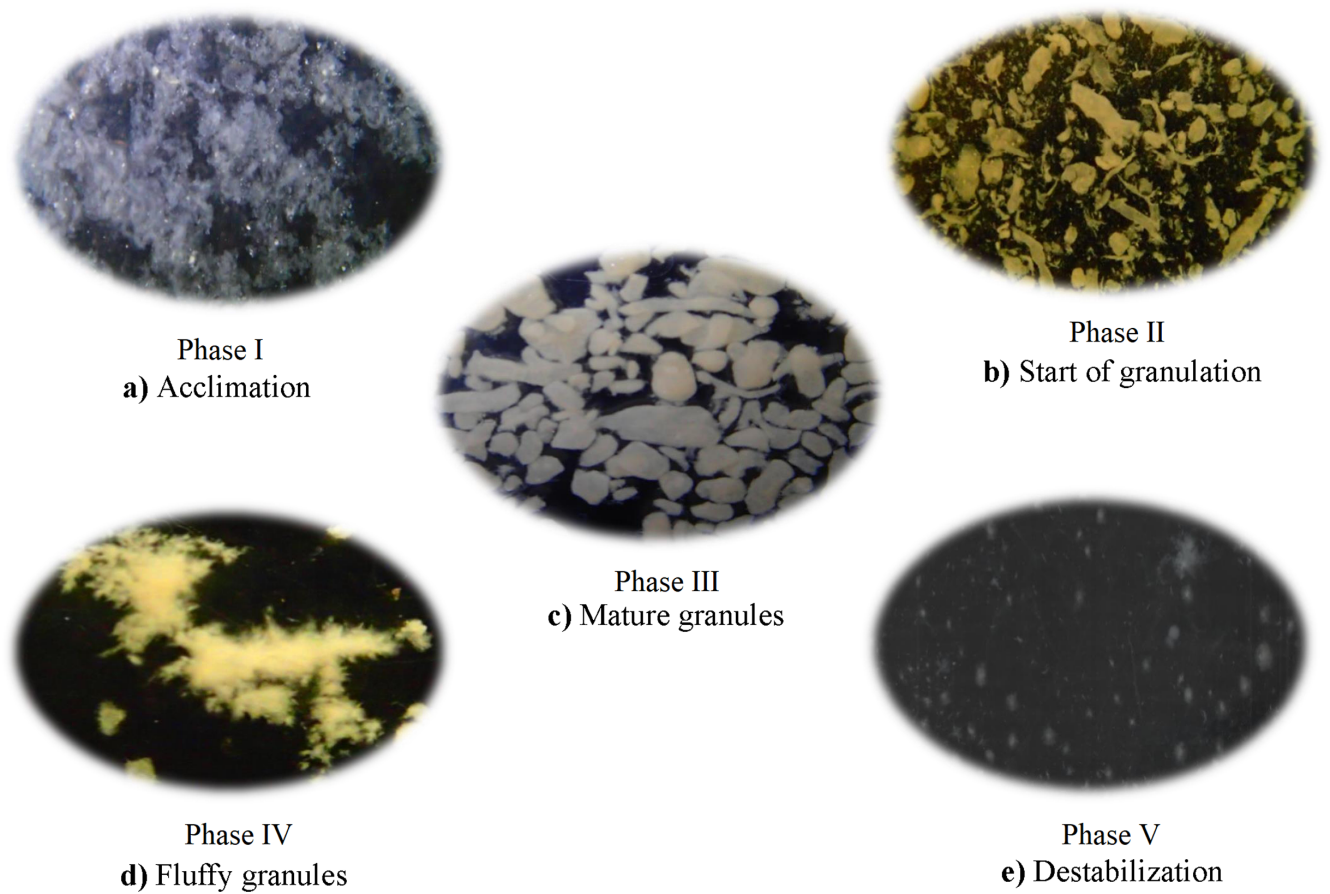
The five phases throughout the Sequencing Batch Reactor operation used in this study; (A) Acclimation, (B) Start of granulation, (C) Mature granules, (D) Fluffy granules and (E) Destabilization.

The phase I or acclimation lasted 12 days and had a settling time of 30 min. In this phase, the sludge had a filamentous morphology (Fig. 5A). The SVI, expressed as COD, decreased from 222.6 in the seed sludge to 70.3 kg/m^3^ at day 12, while the total microbial biomass concentration increased from 3.3 to 7.2 kg/m^3^ (Fig. 1A). The F/M ratio was 2.45 g COD/g MLVSS/day at day 0 and decreased to 1.30 (MLVSS/day) at day 12, while the DO concentration dropped from 4.6 to 1.2 mg/L at day 12 (Fig. 1B). The COD removal efficiency showed little variation during the acclimation phase and was on average 93.0%, but the total N removed increased from 45.0% in the inoculum to 74.0% at day 12 (Fig. 1C).

**Figure 6:**
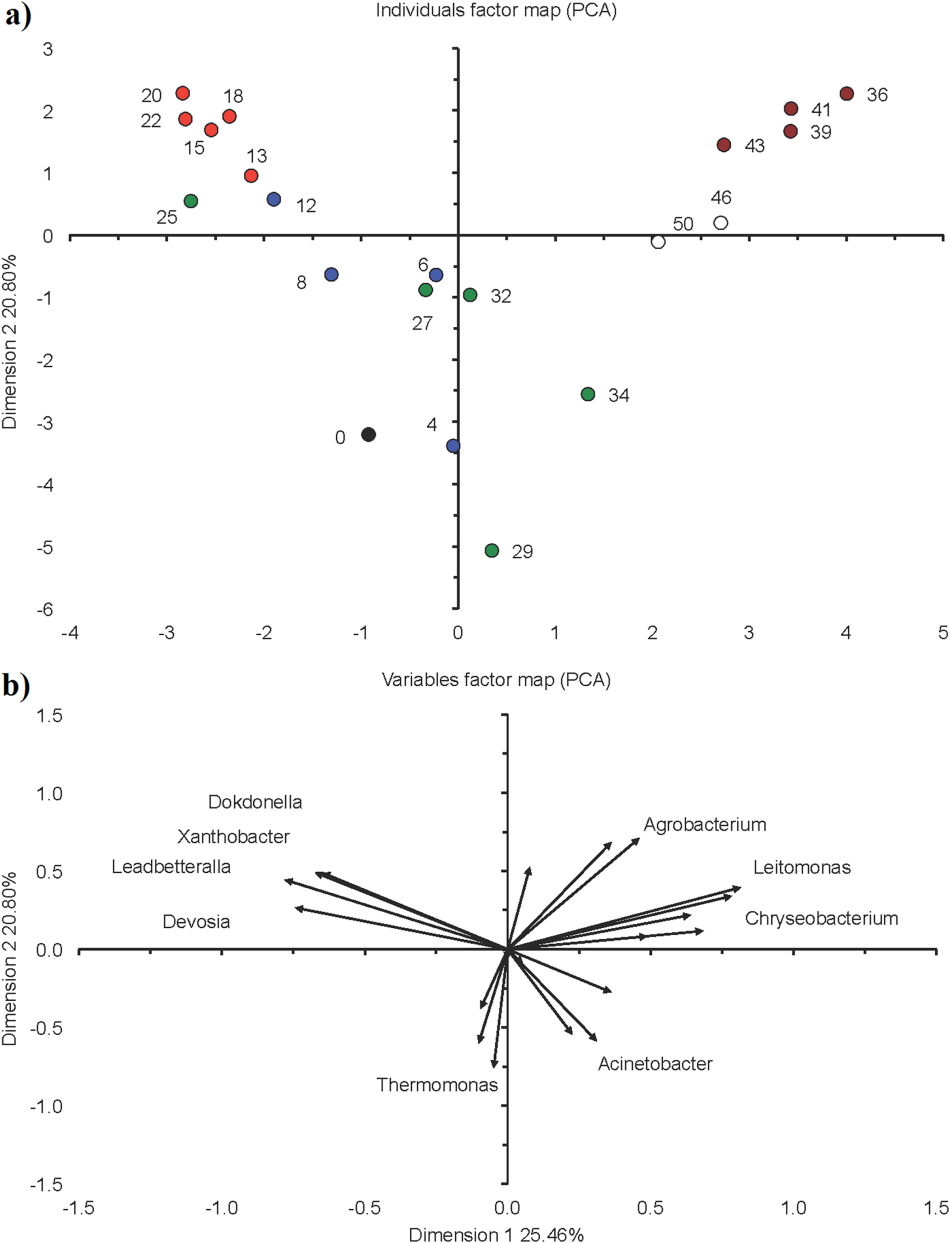
Principal component analysis (PCA) with the 20 most abundant bacterial genera in the sequencing batch reactor used in this study. (A) Individuals factor map and (B) variables factor map. Black circle represents bacterial community structure in the seed sludge (day 0), blue circle in the acclimation phase (day 4, 6, 8, 12), red circle in the start of the granulation phase (day 13, 15, 18, 20, 22), green circle in the granulation phase (day 25, 27, 29, 32, 34), brown circle in the fluffy granule phase (day 36, 39, 41, 43) and white circle in the destabilization phase (day 46 and 50).

During phase I, the relative abundance of *Thiothrix* dropped to 0.7% on day 4, while that of *Acinetobacter* reached a maximum of 70.8% on day 4 (Fig. 2B). The relative abundance of the latter decreased thereafter, but that of *Xanthobacter* increased and reached 42.2% on day 12. Consequently, the bacterial population structure changed clearly in the acclimation phase and after 12 days resembled the bacterial community structure as found at the start of the granulation phase (Fig. 6).

**Figure 7:**
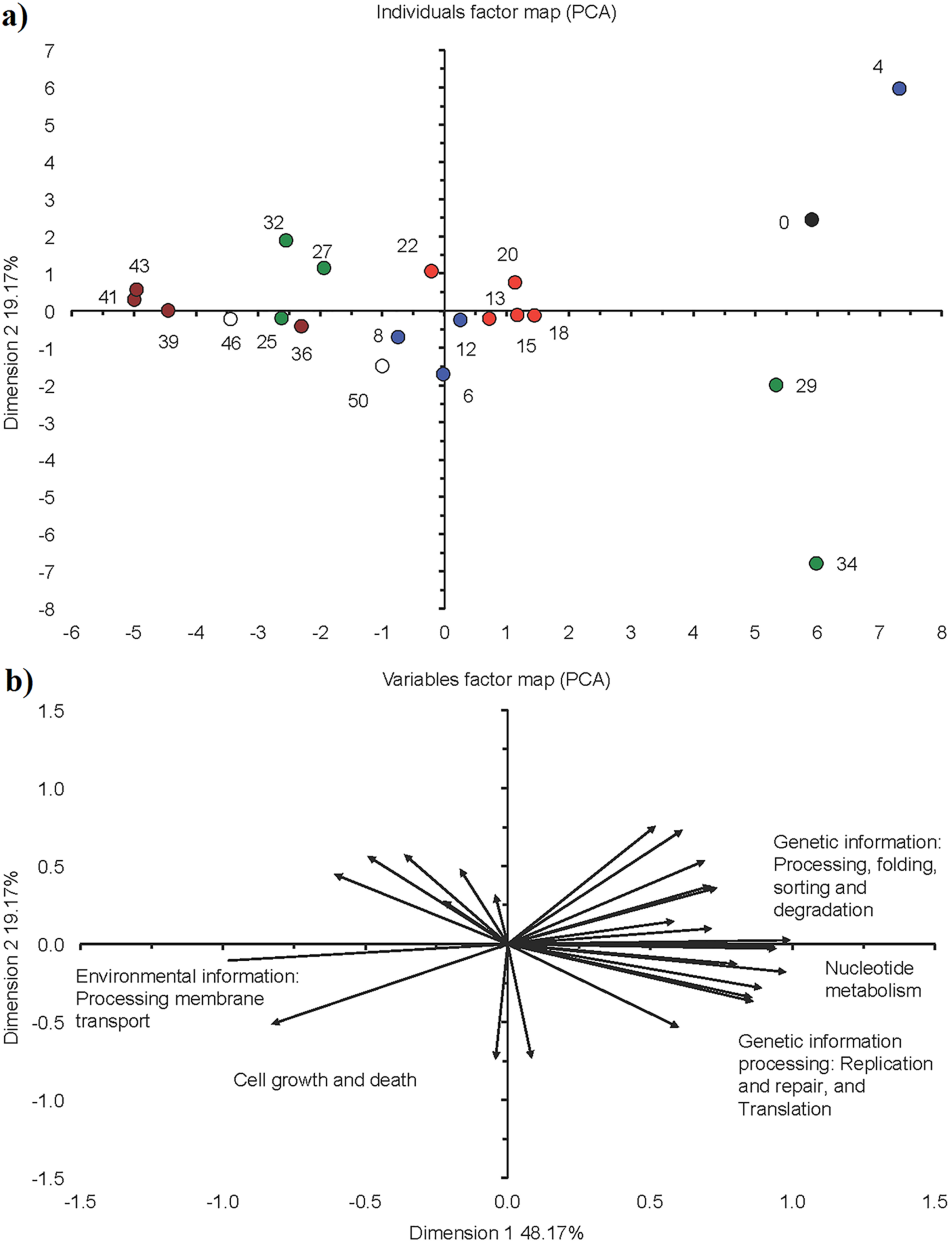
Principal component analysis (PCA) with the functionality of bacterial communities predicted with the Kyoto Encyclopedia of Genes and Genomes (KEGG) as detected in the different phases in a sequencing batch reactor. (A) Individuals factor map and (B) variables factor map. Black circle is bacterial community structure in the seed sludge (day 0), blue circle in the acclimation phase (day 4, 6, 8, 12), red circle in the start of the granulation phase (day 13, 15, 18, 20, 22), green circle in the granulation phase (day 25, 27, 29, 32, 34), brown circle in the fluffy granule phase (day 36, 39, 41, 43) and white circle in the destabilization phase (day 46 and 50).

The comparative metagenomic analysis predicted that 77.3% of the bacteria were possible ammonia oxidizers and 65.5% nitrite reducers during the acclimation phase (mean for days 4, 6, 8 and 12), while 71.5% were possible sulfate reducers and 52.3% had sulfur oxidizing capacity (Table 1). Genetic information of processing, replication and repair decreased toward the end of this phase as predicted by KEGG orthologs, while membrane transport increased (Fig. 3). The predicted KEGG orthologs changed substantially in this phase (Fig. 7). At day 4, the predicted KEGG orthologs were still similar to that in the seed sludge but changed rapidly thereafter as environmental information processing of membrane transport and cell growth and death increased compared to the seed sludge.

In phase I, most phylotypes detected in the seed sludge could still not be assigned to a fungal group phylotypes, although it had dropped to 52.9% at day 12 (Fig. 4). At day 12, the most abundant fungal genus was *Dipodascus*.

### The SBR operation at settling time 3 min: day 13 to day 22: Phase II, start of granulation

In phase II or the start of granulation that lasted from day 13 to day 22, the settling time was changed to 3 min to enhance granule formation (Fig. 5B). In this phase, the slow settling biomass was washed out of the reactor as a result of the decreasing settling time. The biomass was 6.4 g COD/L on day 13 and decreased to <5.7 g COD/L between day 18 and 22. The SVI decreased from 70.3 to 25.7 mL/g and remained constant thereafter indicating that the settling properties of the sludge had improved (Fig. 1A). The DO concentration increased from 1.8 to 4.6 mg/L at day 22, while the remaining F/M ratio was on average 1.4 g COD/g MLVSS/day, which led to the formation of granules with a Feret diameter of 0.9 ± 0.5 mm (median absolute deviation) (Figs. 1B and 5B). The COD removal efficiency showed little variation during the start of the granulation phase and was on average 93.9% (Fig. 2B). The total N removal efficiency further increased to 82.1% on day 22.

On day 13, that is, at the start phase II, the relative abundance of *Xanthobacter* reached 56.6%, but afterwards its relative abundance nearly halved to 28.3% at day 22 (Fig. 2B). The relative abundance of *Devosia*, *Dokdonella* and *Leadbetterella* increased and more than doubled toward the end of this phase. The PCA grouped the bacterial community in the acclimation phase together in the top left quadrant as the relative abundance of *Devosia*, *Dokdonella*, *Leadbetterella* and *Xanthobacter* was generally larger in this phase than in the other phases (Fig. 6).

The comparative metagenomic analysis predicted that during phase II, on average 74.4% of the bacteria (mean for days 13, 18, 20 and 22) had a potential ammonia oxidizing capacity, 59.9% a potential nitrite reducing capacity and 45.1% the potential to fix nitrogen (Table 1). On day 13, the denitrification capacity of the bacteria reached a maximum of 27.9%. On average, 73.1% of the bacteria had the capacity to reduce sulfate and 34.6% to oxidize sulfur during phase II. Most metabolic characteristics as predicted by KEGG orthologs in phase II were similar to those found at the end of phase I (acclimation) and showed little variation (Fig. 3). Consequently, the PCA with the predicted KEGG orthologs grouped the sampling days of this phase close together (Fig. 7).

In phase II, the most abundant fungal genus was *Dipodascus* (50.8%: mean of day 13, 15, 18, 20 and 22), although the relative abundance of *Galactomyces* was high at day 18, that is, 50% (Fig. 4). The relative abundance of the unassigned fungi was still high (mean 30.7%).

### The SBR operation at settling time 1 min: day 25 to day 34: Phase III, mature granules

In phase III (mature granules) (from day 25 to 34), the settling time was reduced to 1 min, the cycling time increased to 6 h, the aeration rate was 2.5 L/min and the substrate concentration three g COD/L, but the OLR was kept constant. As a result, more compact spherical granules were formed with a Feret diameter of 1.3 ± 0.8 mm (median absolute deviation) (Fig. 5C). The SVI remained at 25.6 mL/g, while the biomass was 6.0 g COD/L between day 25 and 32 and increased to 8.0 g COD/L on day 34 (Fig. 1A). The F/M ratio increased from a minimum of 1.2 g COD/g MLVSS/day at day 29 to 1.8 g COD/g MLVSS/day at day 34, while the DO concentration in the reactor decreased from 5.1 at day 27 to 3.5 mg/L at day 34 (Fig. 1B). The COD removal efficiency showed little variation during phase III and was on average 96.0% (Fig. 1C). The N removal efficiency, however, showed large fluctuations. It dropped from 82.1% on day 22 to 56.6% on day 25, increased to 85.8% on day 29 and decreased again to 69.5% on day 34.

Phylotypes belonging to *Acinetobacter*, *Agrobacterium* and *Xanthobacter* were the most abundant genera during phase III, but they showed larger variation over time (Fig. 2B). For instance, the relative abundance of *Acinetobacter* increased from 8.7% at day 25 to 43.3% on day 29, dropped to 5.0% on day 32 and increased again to 55.3% on day 34. Consequently, the bacterial community in phase II was not grouped and gradually changed in structure (Fig. 6). At day 25, the bacterial community still resembled the one found in phase II (start of the granulation), but after 34 days it resembled more the bacterial community in phase IV (fluffy granules) (Fig. 6).

The comparative metagenomic analysis predicted that during phase III, 75.3% of the bacteria (mean for days 25, 27, 29, 32 and 34) had the potential to oxidize ammonia and 67.0% to reduce nitrite (Table 1). The number of possible bacterial nitrogen fixers was 70.5% on day 32, while that of sulfate reducers 80.6% and sulfide oxidizers 64.5%. The predicted KEGG orthologs for cell motility and signaling molecules metabolism were low between day 25 and 34 (Fig. 3). The PCA with the predicted KEGG orthologs showed clear changes at day 29 and 34 compared to days 25, 27 and 32 (Fig. 7). The predicted KEGG orthologs of the latter resembled those of the end of the previous phase, that is, day 25. The predicted KEGG orthologs on day 29 and 34 resembled more those on day 0, that is, seed sludge, and day 4.

In phase II (granulation), the most abundant fungal genera that remained was *Dipodascus* and its relative abundance increased to 90.7% (mean of days 25, 27, 29, 32 and 34) (Fig. 4). Unidentified members of the Agaricomycetes were detected on day 29 only, but no other fungal groups were detected during this phase. The relative abundance of the unassigned fungi was low and only 4.6% (mean of days 25, 27, 29, 32 and 34).

### The SBR operation at settling time 1 min: day 36 to day 43: Phase IV, fluffy granules

Fluffy granules were detected between day 36 and 43, although none of the operating conditions had been altered. The morphology and structural characteristics of the granules were different from those in the previous phases. In the early phases, granules were smaller and more tightly packed than in the later phases when they were large and fluffy (Fig. 5D). The biomass concentration in the reactor that had reached a maximum of 8.0 g COD/L at the end of phase III, mature granules, that is, day 34, decreased to 3.1 g COD/L on day 43, that is, the end of phase IV, fluffy granules (Fig. 1A). The SVI increased from 28.9 mL/g on day 36 to 47.6 mL/g on day 43. The F/M ratio increased from 1.7 g COD/g MLVSS/day on day 36 to 2.6 g COD/g MLVSS/day on day 43, while the DO showed little variation (Fig. 1B). During this phase, both the mean COD and N removal efficiency showed little variation (Fig. 1C).

*Agrobacterium* was the dominant bacterial genus during phase IV (mean 28.7%), followed by *Acinetobacter* (18.7%) (Fig. 2B). The relative abundance of *Xanthobacter* increased sharply from 0.3% on day 36 to 19.0% on day 43. However, the bacterial community in phase IV was similar and grouped together in the top right quadrant (Fig. 6). The relative abundance of *Agrobacterium*, *Leitomonas* and *Chryseobacterium* was larger in this phase than in the other phases.

The comparative metagenomic analysis predicted that 71.8% of the bacteria (mean for days 36, 39 and 43) had the capacity to oxidize ammonia in phase IV and 63.8% to reduce nitrite. The nitrogen fixing capacity was 57.5% between day 36 to 43 and the sulfate reducing capacity 71.6% (Table 1). The predicted KEGG orthologs were similar in this phase (Fig. 3). Compared to the previous phase, that is, phase III, mature granules, the predicted KEGG orthologs, environmental information processing of membrane transport and cell growth and death increased further in phase IV (Fig. 7).

In phase IV, *Dipodascus* remained the most abundant fungal genera (88.8% mean of day 36, 39, 41 and 43) (Fig. 4). Unidentified members of the Agaricomycetes were during most of this phase (mean 4.8%), but no other fungal groups were detected during this phase. The relative abundance of the unassigned fungi remained low (mean 4.8%).

### The SBR operation at settling time 1 min: day 46 and day 50: Phase V, destabilization

Granules destabilized quickly in this phase that lasted from day 46 to 50. The granules with filamentous overgrowth were not stable and were washed out of the reactor within a few days (Fig. 5E). The SVI increased sharply and reached 187.6 mL/g on day 50 while the biomass concentration dropped to 2.8 g COD/L (Fig. 1A). Both the COD and N removal efficiency showed little variation during this phase (Fig. 1C).

Members of *Xanthobacter* dominated with a relative abundance of 13.0% on day 46, but at day 50 phylotypes belonging to *Acinetobacter* (32.3%) dominated (Fig. 2B). The bacterial community structure was still similar to that in phase IV (Fig. 6).

The mean relative abundance of *Dipodascus* was 88.4% and that of *Agaricomycetes* 6.7% (Fig. 4). During phase V, the comparative metagenomic analysis predicted that 80.3% (mean of days 46 and 50) of the bacteria were possible ammonia oxidizers, 68.5% nitrite reducers and 75.7% sulfate reducers while the number of nitrogen fixers, sulfide oxidizers and sulfur oxidizers had decreased compared to phase IV (Table 1).

The predicted KEGG orthologs were similar at day 46 and 50 (Fig. 3). Compared to the previous phase, that is, phase IV (fluffy granules), the predicted KEGG orthologs, the environmental information processing of membrane transport and cell growth and death, decreased compared to days 39, 41 and 43 of phase IV (Fig. 7).

## Discussion

### Seed sludge

The inoculum had a SVI value of 222.6 mL/g, which is characteristic for activated sludge. A SVI value >150 mL/g is commonly associated with a filamentous morphology (Crites & Technobanoglous, 1998). Phylotypes belonging to the *Gammaproteobacteria* (mostly *Thiothrix*) and *Alphaproteobacteria* (mostly *Rhodobacter*) were the most abundant bacterial phyla in the seed sludge and they dominate often in conventional activated sludge (Hai et al., 2014). Members of *Thiothrix* are filamentous sulfur-oxidizing bacteria that can contribute to filamentous sludge bulking (Kanagawa et al., 2000). Phylotypes of *Thiothrix* are enriched in wastewaters with high OLRs, large amounts of low molecular weight fatty acids, high concentrations of reduced sulfur compounds, and low DO and nutrient content (Henriet et al., 2017). The seed sludge with its high SVI content was an ideal environment for members of this genus.

Members of *Rhodobacter*, are Gram-negative, often photosynthetic, metabolically diverse and they can grow in salt and nutrient rich environments, such as anoxic waters, soil, sediment and sludge (Wilkinson et al., 2011; Lin et al., 2015). They are versatile and often abundant in wastewater sludge as found in this study (Hai et al., 2014; Ye et al., 2016).

### The SBR operation at settling time 30 min: day 0 to day 12: Phase I, acclimation phase

In the same type of reactor, the SVI normally decreases while the biomass increases as found in this study (Beun, Van Loosdrecht & Heijnen, 2002). Li, Li & Yu (2011) stated that a F/M ratio >1.1 g COD/g MLVSS/day facilitates changes in the bacterial community in a reactor, as detected in this study.

The relative abundance of phylotypes belonging to *Acinetobacter*, which account for 1–10% of the bacterial community in activated sludge (Hiraishi, Masamune & Kitamura, 1989) and 4.3% in the seed sludge in this study, increased more than 15 times on day 4. The use of acetate as sole C substrate enriched them as members of *Acinetobacter* prefer acetate as substrate, and convert and store it as polyhydroxyalkanoates (Rustrian, Delgenes & Moletta, 1996). The change in C substrate reduced the relative abundance of *Thiothrix* strongly at day 4 and they remained mostly undetectable in the reactor afterwards.

Members of *Acinetobacter* were gradually replaced as the most abundant bacterial genus by phylotypes belonging to *Xanthobacter* during the acclimation phase, which are important participants in the N cycle. Strains of *Xanthobacter autotrophicus* have been isolated from a submerged fixed-film reactor with denitrifying capacity and they reduce nitrate or nitrite to nitrous oxide or dinitrogen (Gómez et al., 2005). Some species of *Xanthobacter* fix nitrogen and some produce extracellular polymeric substances under harsh conditions (Wiegel, 2006).

### The SBR operation at settling time 3 min: day 13 to day 22: start of granulation

On day 12, the settling time was reduced to stimulate the formation of granules. It is known that under stronger hydraulic selection pressure (settling time), microorganisms adapt to avoid being washed out from the reactor through microbial self-agglomeration (Qin, Liu & Tay, 2004). The SVI value remained at 25.7 mL/g during this period. Zheng, Yu & Sheng (2005) obtained similar SVI values of 23 mL/g for granular sludge. The F/M decreased to 1.3 g COD/g MLVSS/day on day 12, which facilitated the formation of large granules. Granules formed at a F/M ratio of 1.1 g COD/g MLVSS/day were observed to have a higher protein proportion in the extracellular polymeric substances (Jiang et al., 2004).

The relative abundance of *Agrobacterium* had increased toward the end of phase I (acclimation) and further increased in this phase. *Agrobacterium* sp. ZX09 is a salt tolerant strain that produces extracellular polymeric substances (Xiu et al., 2010). As such, members of *Agrobacterium* could participate in aggregation through the excretion of these extracellular polymeric substances. Additionally, the *N*-acylated homoserine lactone system, which is part of the quorum sensing system and found in Gram-negative bacteria like *Agrobacterium*, has been shown to control biofilm formation (Goh, Rice & Kumar, 2005).

Dokdonella are Gram-negative *Proteobacteria* and their genome sequences contain genes for the formation of adhesins (Moreira et al., 2004) so they can attach to a surface enhancing their resistance to biotic and abiotic stress (Danhorn & Fuqua, 2007). Their capacity to produce adhesins might explain the high relative abundance of this genus toward the end of the start of phase I (granulation).

### The SBR operation at settling time 1 min: day 25 to day 34: phase III, mature granules

A recent study reported that the polysaccharides/proteins ratio correlated inversely with the settling time, that is, a shorter settling time stimulated the yield of polysaccharides (Li, Li & Yu, 2011). The compact granules formed at this phase had a low SVI value (22.3 mL/g), similar results were obtained for compact granules in an acetate fed reactor (Carucci et al., 2009). The SVI of the granular sludge showed a positive correlation with surface hydrophobicity. The lower F/M ratio (mean = 1.4 g COD/g MLVSS/day) could also contribute to the formation of the smaller granules as reported by Li, Li & Yu (2011). The compact granules became more efficient in the removal. The COD removal was 95% and N 85% on days 29 and 32. Compact granules have extended surface areas that allow faster nutrient uptake and substrate mineralization.

The bacterial genera that dominated during phases I and II, that is, *Acinetobacter*, *Xanthobacter* and *Agrobacterium* continued to dominate the bacterial population in phase III. *Acinetobacter calcoaceticus* has been found at 200–250 μm beneath the surface of aerobic granules with an extracellular polymeric substances layer protecting them against phenol toxicity (Adav & Lee, 2008), while *Xanthobacter* produces extracellular polymeric substances and contributes to the formation of more compact and stable granules (Kichemazova et al., 2017).

Two other bacterial genera were enriched during phase III, *Delfia* and *Rhodobacter*. *Comamonadaceae* to which *Delfia* belongs, dominate often in bacterial communities of a SBR (Ge, Batstone & Keller, 2013) and *Delftia acidovorans* is a non-fermentative Gram-negative *Betaproteobacteria* that produces biofilm (Liu et al., 2016). Phylotypes belonging to *Rhodobacter* have been found in the outer layer of a mature granule (Mieczkowski et al., 2016). They produce extracellular polymeric substances so probably participated in the formation of the outer layers of a mature granule (Sheng, Yu & Wang, 2006).

*Dipodascus* belongs to the Saccharomycetales and is described as a highly filamentous species (Weber et al., 2007). The high relative abundance of *Dipodascus* mostly from the end of phase II onwards, indicates that its members might be part of the internal structure of the compact granules as reported by Weber et al. (2007). Microscopic analyses of mature granules showed that their inner part contained remnants of hyphae. The relative abundance of Ascomycota found in this experiment was higher than that of Basidiomycota. Maza-Márquez et al. (2016) reported Saccharomycetes with a relative abundance of up to 75% in samples taken from a membrane bioreactor fed with urban wastewater.

### The SBR operation at settling time 1 min: day 36 to day 43: Phase IV, fluffy granules

The increase in the F/M ratio between day 36 and 43 could be related to the formation of oversized granules as it has been reported that a higher F/M ratio produced larger granules (Li, Li & Yu, 2011). At the end of the previous phase, the concentration of biomass increased together with a decrease in the minimum DO concentration. Oxygen gradients form anaerobic microsites within aerobic granules as oxygen diffusion is restricted and substrate diffusion limitations in the core of the granules. This might have induced the growth of filamentous structures (Martins, Heijnen & Van Loosdrecht, 2003).

After some time, however, the granules become less stable (Wan et al., 2014) as filamentous bacteria can overgrow them (Liu & Liu, 2006). Although phylotypes belonging to *Rhodobacter* participate in granule formation they have also been found in disintegrated granules so they might participate in their disintegration (Wan et al., 2013). Factors that affect the stability of the granules are low pH (Wan et al., 2014), substrate availability, DO concentration, retention of solids in the bioreactor, nutrient deficiency and temperature (Liu & Liu, 2006).

*Flavobacterium* (*Bacteroidetes*) are often abundant in activated sludge and they produce polymeric substances (Preut, 2014). Phylotypes belonging to *Flavobacterium* have been found on the surface of mature aerobic granules and some *Flavobacterium* spp. species agglomerate with various Gram-negative and Gram-positive bacteria (Basson, Flemming & Chenia, 2008). Their high relative abundance at the beginning of phase IV, fluffy granules, however is difficult to explain as they appear to participate in the disintegration of the granules.

The fluffy appearance of the granules could be due to the presence of *Dipodascus* as members of this genus are highly filamentous, or by an overgrowth of filamentous bacteria. Nevertheless, the importance of bacterial groups with a low relative abundance should not be neglected, as some members of the Cytophagaceae (relative abundance of 2.22%) have been identified as filamentous bacteria (Basson, Flemming & Chenia, 2008).

### The SBR operation at settling time 1 min: day 46 to day 50: phase V, destabilization

The reactor operation remained the same, and phylotypes belonging to *Xanthobacter* dominated the bacterial community structure in the SBR. Members of *Rhodobacter* have been found in degenerated granules and they could be involved in granule destabilization (Wan et al., 2013). Wan et al. (2013) showed that a decrease in cyclic diguanylate (c-di-GMP) coincided with a decline in the amount of polymeric substance in the granules that led to granule disintegration. *Dipodascus* could also have been involved in the destabilization of the granules as filamentous cells have been reported to be detrimental for reactor operation causing bulking (Martins et al., 2004).

No Archaea were detected in the SBR reactor. Jiang et al. (2004) also reported the absence of Archaea in aerobic granules.

### Comparative phenotypic analysis of bacterial communities in the SBR

The predicted ammonia oxidizing, nitrite reducing, sulfate reducing and sulfur oxidizing activity increased at the beginning of phase I (acclimation) compared to the seed sludge while nitrogen fixation and sulfide oxidizing activity decreased. The increase in predicted nitrite reducing activity could be due to an increase in the relative abundance of *Rhodobacter*. Nitrite reductase enzyme has been found in the cytosol and periplasm of *Rhodobacter capsulatus* (Martínez-Luque, Dobao & Castillo, 1991). The increase in predicted sulfur and sulfide oxidizing activities could be due to an increase in the relative abundance of *Acinetobacter*. Some strains of Acinetobacter oxidize elemental sulfur (S) to thiosulfate (S_2_O_6_^2−^) (Subhraveti et al., 2017). The predicted ammonia oxidizing, nitrite reducing and sulfate reducing activity showed only small variation over time in the SBR. At phase II, start of granulation, an increased predicted nitrogen fixation capacity was detected that could be due to the increased relative abundance of *Xanthobacter*. Members of *Xanthobacter* are able to fix nitrogen (Martins, Heijnen & Van Loosdrecht, 2003). The maximum denitrifying activity predicted on day 13 could be related to the presence of *Agrobacterium*. Phylotypes belonging to this genus are denitrifiers (Merzouki et al., 1999). Afterwards, the relative abundance of *Agrobacterium* and the predicted denitrifying activity decreased in the SBR.

At day 32, that is, during the mature granule phase, predicted nitrogen fixation reached a maximum as did the relative abundance of *Xanthobacter*. Phylotypes belonging to this genus can grow chemolithoautotrophically and use molecular nitrogen (N_2_) when favorable H_2_ concentration are provided and O_2_ partial pressure <5% (v/v) (Martins, Heijnen & Van Loosdrecht, 2003). During the fluffy granule phase, predicted nitrogen fixation decreased, which correlated with a decrease in the relative abundance of *Xanthobacter*. Similarly, the relative abundance of *Acinetobacter* dropped, explaining the reduction of the predicted sulfur and sulfide oxidizing activity.

### Predicted KEGG orthologs of bacterial communities in the SBR

During phase I, the predicted KEGG orthologs were mostly related to signaling molecules, amino acid and glycan synthesis, folding and motility. The homoserine lactone system allows bacteria to monitor their own population density and start preparing for adhesins synthesis (Whitfield & Valvano, 1993). Adhesion is a conserved virulence mechanism found in Gram-negative bacteria, which is the first step in biofilm formation. Adhesins must pass through β-helical membrane transporters to become active. During this process they fold into their final conformation. Adhesins can also recognize glycan structures on their ligands and mediate cell-cell interactions. The interaction between the prokaryotic cytoskeleton and cell adhesion sites leads to cell locomotion along the substrate (Van den Ent, Amos & Löwe, 2001).

At the start of granulation (phase II), activity was related to membrane transport and cell growth, which correlate with an increase in granule size, while in phase III, mature granules, it was related mainly to carbohydrate metabolism and transcription. Bacteria must adapt their metabolic activity to the nutrients available through the induction of specific catabolic operons induced often by the substrate; in this case acetate (Deutscher, Francke & Postma, 2006). In phase IV (fluffy granules), signal transduction, cell motility and transcription activity were dominant. This could be explained by cell migration that is controlled by intracellular biochemical signals (Mitra, Hanson & Schlaepfer, 2005). In phase V (destabilization), signal transduction and cell motility were dominant again. This suggests that bacteria respond to the stress, sensing the media through signaling molecules to adapt to the changing environment (Rieu et al., 2008).

## Conclusions

The SBR reactor was operated at decreasing settling times that promoted the formation of granules. The SBR operation can be divided into five phases defined by the characteristics of the granules. On day 12, that is, in phase I, the acclimation, the biomass showed a rapid increase. Members of the Gammaproteobacteria were the most abundant bacterial class with most phylotypes belonging to *Acinetobacter*. On day 15, at phase II, start of granulation, *Agrobacterium* reached its maximum relative abundance indicating its participation in cell aggregation. Once the granulation was complete, the SVI reached a minimum with dense and stable granules (phase III). The relative abundance of *Xanthobacter* reached a maximum between days 29 and 32 and its members might have had a positive effect on nitrogen removal. From day 36 to 43, fluffy granules (phase IV) were found in the reactor presumably as a result of limited DO and nutrient diffusion inside the granules that induced the growth of filamentous structures. During phase V, the destabilization, Rhodobacteraceae reached their maximum relative abundance suggesting members of this family could be involved in granule disintegration. However, the predicted functionality of most of the microbial groups found in this experiment remained difficult to explain. No Archaea were detected in the SBR with the primers that were used in this study, but the Fungi *Dipodascus* and Ascomycetes were detected from phase III until the end of the reactor operations. Further studies could be done to determine the effect of higher organic loads and different oxygen concentrations on the microbial populations as concentration gradients seem to be the variables that most affected the stability of the granules. Additionally, it would be interesting to conduct research to understand the function of each microbial group so as to develop strategies that improve granules stability and SBR performance.

## Supplemental Information

10.7717/peerj.7152/supp-1 Supplemental Information 1 Nearest sequenced taxon index (NSTI) scores from samples obtained during the sludge granulation process in a sequencing batch reactor against the KEGG database.

10.7717/peerj.7152/supp-2 Supplemental Information 2 Raw data for biomass concentration, sludge volume index, Food to mass ratio, minimum DO observed during each cycle in the reactor and COD and N removal.

## Additional Information and Declarations

### Data Availability

The following information was supplied regarding data availability: The datasets supporting the conclusions of this article are available in NCBI’s SRA, BioProject accession numbers PRJNA419221 and PRJNA422681. Raw data is available as a Supplemental File.
