# Supplementary material for: Microbial community dynamics during aerobic granulation in a sequencing batch reactor (SBR)
Source: PeerJ. 2019 Aug 29;7:e7152. doi: 10.7717/peerj.7152 (PMC6717656; doi:10.7717/peerj.7152)
Supplement: Supplemental Information 1 [file peerj-07-7152-s001.pdf]

Table S1. Nearest sequenced taxon index (NSTI) scores from samples obtained during the sludge granulation process in a sequencing batch reactor against the KEGG database.

Day	Sample	NSTI value	Phase
0	T0.1	0.1337	Seed sludge
	T0.2	0.1333	
	T0.3	0.1295	
4	T1.1	0.0670	Acclimation
	T1.2	0.0645	
	T1.3	0.0554	
6	T2.1	0.1259	
	T2.2	0.1224	
	T2.3	0.1250	
8	T3.1	0.1584	
	T3.2	0.1457	
	T3.3	0.1518	
12	T4.1	0.0598	
	T4.2	0.0615	
	T4.3	0.0610	
13	T5.1	0.0621	Start of granulation
	T5.2	0.0646	
	T5.3	0.0505	
15	T6.1	0.0572	
	T6.2	0.0598	
	T6.3	0.0557	
18	T7.1	0.0509	
	T7.2	0.0556	
	T7.3	0.0584	
20	T8.1	0.0654	
	T8.2	0.0733	
	T8.3	0.0812	
22	T9.1	0.1072	
	T9.2	0.1094	
	T9.3	0.1051	
25	T10.1	0.0973	Mature granules
	T10.2	0.1000	
	T10.3	0.1051	
27	T11.1	0.1009	
	T11.2	0.1107	
	T11.3	0.1143	
29	T12.1	0.1106	
	T12.2	0.0959	
	T12.3	0.0894	

Table S1. Continued.

32	T13.1	0.0981	
	T13.2	0.0882	
	T13.3	0.0825	
34	T14.1	0.0930	
	T14.2	0.0975	
	T14.3	0.1045	
36	T15.1	0.0817	Fluffy granules
	T15.2	0.0861	
	T15.3	0.0950	
39	T16.1	0.0844	
	T16.2	0.0891	
	T16.3	0.0956	
41	T17.1	0.0847	
	T17.2	0.0863	
	T17.3	0.0837	
43	T18.1	0.0753	
	T18.2	0.0772	
	T18.3	0.0687	
46	T19.1	0.0789	Destabilization
	T19.2	0.0787	
	T19.3	0.0782	
50	T20.1	0.0748	
	T20.2	0.0606	
	T20.3	0.0650	
